# Consumption of Noncommercial Alcohol among Alcohol-Dependent Patients

**DOI:** 10.1155/2013/691050

**Published:** 2013-04-22

**Authors:** Y. E. Razvodovsky

**Affiliations:** Grodno State Medical University, Street Gorky 80, 230009 Grodno, Belarus

## Abstract

This study explores types of alcohol and surrogates consumed, patterns of consumption, and reasons behind noncommercial alcohol consumption among alcohol-dependent patients in Belarus. The study was conducted in the Belarusian city Grodno in 2012 with 223 alcoholics admitted to narcological clinic using structured interviews. The results suggest that at least 20.2% of alcohol dependent patients regularly consume samogon and 11.8% of patients use surrogates, the most popular among which are medications with a high percentage of ethanol and industrial spirits. The belief that, according to quality criteria, samogon exceeds licensed vodka is the main motive for its consumption. The results of this study suggest the existence of the problem of consumption of noncommercial alcohol among alcohol dependent patients in Belarus.

## 1. Introduction

 Noncommercial alcohol has recently become the subject of much attention from alcohol policy experts [1, 2]. The problem of the consumption of noncommercial alcohol in the Commonwealth of Independent States (CIS) countries has attracted the attention of researchers and specialists in the public healthcare field after the epidemic of poisonings by the so-called surrogate alcohols, which swept across Russia and Belarus in 2006 [3–5]. In the second half of 2006, about 11 thousand people in 22 regions of Russia came to hospitals with symptoms of poisoning by alcohol surrogates [3]. During forensic chemical analysis of material from the corpses of those who died as a result of poisoning by surrogates in Russia in 2006, the presence of higher alcohols and their esters and glycols (propanol, isopropanol, butanol, isobutanol, isopropanol, and acetone) was discovered [6, 7]. The main macromorphological manifestations of poisonings by substitutes include severe dystrophic changes in all the internal organs (except the adrenal glands) with predominant damage to the liver and kidneys, which was confirmed by the corresponding clinical picture with development in the majority of victims of jaundice with the presence of bile pigments in the lumen of the renal tubules, detected by histological examination [8]. 

 Despite the extreme urgency of the problem, our knowledge with respect to the prevalence of the consumption of surrogates in CIS, as well as the style and motives of their consumption, remains fragmented [9–13]. That said, knowledge of the social and epidemiological correlates of this phenomenon is a necessary condition for the development of a prevention strategy. Isolated studies devoted to this problem suggest that the main consumers of noncommercial alcohol are heavy drinkers, that is, persons who abuse alcohol and also suffer from alcohol dependence [14]. 

In a recent study conducted in Novosibirsk, Russia, various aspects of the problem of consumption of surrogates among substance abuse clinic patients, including types of surrogates consumed, the pattern of their consumption, and the reasons for their consumption, were studied [15]. It turned out that all the patients periodically use industrial spirits, medications containing alcohol (Hawthorn tincture), antiseptics, and eau de cologne. The main reason for the popularity of surrogates among people dependent on alcohol is their physical and financial availability, since diluted industrial alcohol is 3 times cheaper than the cheapest vodka. Based on this, the authors conclude that the main reason for the prevalence of the consumption of substitutes is the high price of licensed vodka. 

The main source of undocumented alcohol in Belarus is samogon (homemade vodka). Noncommercial alcohol in Belarus includes samogon, counterfeit vodka, industrial alcohol, alcohol-containing medicinal preparations, and cologne [11]. Samogon is produced both in homes and in remotely located minifactories that sell to others. In 2009, 1.8 million liters of alcohol-containing liquid were seized in Belarus from illegal sales, and more than 2,000 minifactories producing samogon were shut down [5]. Many households also make homemade wine by fermenting the juice of various fruits and berries. However, this type of noncommercial alcohol does not make up a significant share in the structure of undocumented alcohol consumption [11]. 

To date, systematic studies devoted to the prevalence of illegal alcohol and surrogates consumption among the population of heavy drinkers in Belarus have not been conducted. The goal of this pilot study was to some extent to fill this gap by studying the prevalence, drinking pattern, types, reasons, and correlates of the consumption of noncommercial alcohol among alcohol abuse clinic patients. 

## 2. Materials and Methods

Taking part in the study were 223 alcohol-dependent men, who were treated in the alcohol abuse treatment department of the Grodno Regional Psychoneurologic Dispensary (Belarus). Data was collected by trained interviewers through structural face-to-face interviews in the respondents' wards and lasted approximately 30 minutes. The questionnaire covered a range of characteristics including socioeconomic and demographic variables, drinking frequency of all beverages types including noncommercial alcohol, the amount of each beverage type drunk on a usual occasion defined in quantity units commonly used (bottles of beer and grams of wine and vodka), information about health problems arising from the consumption of alcohol with emphasis on noncommercial alcohol (hangover and poisoning), the motives that guided responders in their choice of alcoholic beverages, the sources of noncommercial alcohol, and the opinion of responders regarding the quality of licensed alcohol and noncommercial alcohol. For nonbeverage alcohol (surrogate) only frequency of consumption was asked since there are no standard measures of volume consumed. The statistical analysis for the information from structured interview was conducted using Microsoft Excel where the data was tabulated and evaluated.

## 3. Results 

### 3.1. Description of the Sample

Selected sample characteristics are summarized in Table 1. The average age of the patients was 39.7 ± 1.2 years. 43.2% of patients were married and 56.8% were single. The patients were distributed by level of education as follows: primary school –3.2%, middle school –77.4%, and higher education –19.5%. Distribution according to social status was as follows: blue collar workers –51.8%, unemployed –33.3%, white collar workers –8.7%, and retirees –6.2%. The income level of 43.3% patients was below average, of 34.8% was average, and of 22.0% was above average. 

### 3.2. Types of Alcohol Consumed and Patterns of Consumption

The results of the survey showed that 40.8% of patients prefer vodka, 37.3% consume vodka and fortified wines, and 39.2% consume vodka and beer. According to the survey results, most patients (62.7%) drink alcohol 2 to 3 times a week, and 18.9% do so daily. The average level of alcohol consumption was 31.2 liters per year (in terms of absolute alcohol), which is a rather high number. According to the results of the survey, 30.5% of patients usually consume 300–500 mL of vodka, and 33.2% drink more than 500 mL of vodka during a single drinking occasion. 

The motives in the choice of alcoholic beverage were as follows: quality –44.3%, physical accessibility –26.3%, cheapness –15.5%, and “lethal effect” –9.3%. Contrary to expectations, more than half of the patients when choosing an alcoholic beverage are guided by quality criteria. The presence of the hangover syndrome was acknowledged by 89.2% of patients, which may indirectly attest to the significant degree of their candor in answering the questions asked. 

### 3.3. Prevalence and Reasons for Drinking Noncommercial Alcohol

The most popular unlicensed alcoholic beverage among persons dependent on alcohol is samogon, which 52.9% of patients consume at least one time a month while 14.3% do so at least one time a week and 8% do so 2-3 times a week and 2.1% on a daily basis. 

As a rule, respondents are inclined to hide the fact of consumption of prohibited substances by them and are more likely to admit that their friends use these substances. According to the survey, 75.1% of patients indicated that they have friends who consume samogon, and 29.2% know those who make samogon. This data indirectly indicates a significant prevalence of the consumption of samogon among the population. 

Answers to the question regarding the reasons for consuming moonshine are distributed as follows: “samogon is chemically a purer product than licensed vodka” –41.4%, “the cheapness of samogon” –27.6%, “samogon is a traditional alcoholic beverage” –18.4%, and “physical availability of samogon” –10.3%. 

One of the types of unlicensed alcohol is homemade wine, which is made from various fruits. However, homemade wine has little real weight in the structure of unrecorded consumption of alcohol, since only one-third (34.3%) of patients consume it, with 22.7% of them consuming wine once a month or less and only 2.8% consuming it daily. 

According to the results of the survey, 11.8% of patients use surrogates (industrial spirits and medications containing alcohol: Hawthorn and Motherwort tinctures). More than one-third (36.2%) of the patients admitted that they had to buy countrified vodka without excise stamps “on the sly.” A significant proportion of patients (36.2%) indicated that their friends consume surrogate alcohols. According to respondents, the most common alcohol surrogates are medicinal tinctures with high volume of ethanol purchased in pharmacies and industrial spirits, which is bottled at home and sold under the guise of licensed alcohol. Medications with a high percentage of ethanol are used because they are regarded as a “pure medicinal product.” 

### 3.4. Perceived Quality of Noncommercial Alcohol and Impact on Health

The majority of patients (77.4%) believe that moonshine is a chemically pure, “natural” product, and only 22.6% believe that moonshine is a chemically “dirty” product, hazardous to health. A representation of the high quality of moonshine could be made on the basis of personal experience of consumption because about half of the respondents (45.7%) had not experienced any problems with their health after consuming moonshine. At the same time, 18.8% of patients noticed symptoms of poisoning (nausea and vomiting), and 34.1% felt unwell after consuming moonshine. Conflicting data regarding the effects of alcohol consumption on health may be explained by the differences in the quality of moonshine. The majority of respondents noted that the quality of moonshine, produced for personal use, is significantly better than that produced for sale. 

Only a small number of patients who consume moonshine (9.8%) make moonshine themselves. The majority acquire it on “site” (47.8%) or from friends (42.4%). It is assumed that moonshine acquired from friends has a better quality than that acquired “on site” (private apartment and houses). 

During the interview, many patients said that they know of cases when counterfeit vodka, made from industrial alcohol, was sold in government trade shops. This is probably why 74.2% of patients indicated that, after they consumed low-quality vodka bought in government trade shops, the signs of poisoning were noted. Many respondents attributed having a severe hangover to the bad quality of licensed and countrified vodka. 

### 3.5. Perception of Policies on Noncommercial Alcohol

 It should be noted that many patients reported a decrease in the availability of samogon and surrogates in recent years. According to them, samogon and surrogates were able to be freely obtained earlier at “sites,” which worked around the clock, but recently this has become more problematic. The main reason for the reduction in the availability of unlicensed alcohol, according to the patients, is the enforcement of the efforts against the illegal circulation of alcohol in Belarus. 

Of interest is the presumptive behavior of alcohol-dependent patients with a change in licensed alcohol affordability. To the question “What will you do if you lack financial resources?” 41.6% of patients answered that they will stop drinking, 31.6% will switch to cheaper alcoholic beverages, and 25.8% will drink the expensive and high-quality alcoholic beverages, but in smaller amounts. To the question “What will you do if the state vodka gets more expensive?” 42.0% of the patients answered that they will start to drink less, 21.8% will start to drink fortified fruit and berry wines, 13.8% will start to drink beer, and 19.0% will drink moonshine. None of the patients intend to drink surrogates if vodka becomes more expensive. These data indicate that the main potential alternative to vodka is fortified wine and moonshine. 

## 4. Discussion

In fact, this is the first in-depth study of the alcohol and surrogates drinking pattern, types, reasons, and correlates among alcohol dependent persons in Belarus. According to the results of the survey, vodka is the product of first choice for persons dependent on alcohol in the Belarus sample. Fortified wines are also popular and generally consumed without meals in order to achieve a rapid intoxication effect. The popularity of fortified wines is due to their relative cheapness, as 0.5 liters of wine with strength of 18–20° costs about 1$. The results of the survey suggest that the majority of patients have a high risk of “acute” alcohol-related problems, the threshold level for which is considered a dose of 40 grams (in terms of absolute alcohol), equivalent to 100 mL of vodka [16–18]. It should be noted that the pattern of beverage types consumed by alcohol dependent patients is similar to that of the general population, when their patterns in terms of units of alcohol per drinking occasion are more detrimental [5]. 

The findings from preset study showed that it was relatively common for alcohol dependent patients to drink samogon and surrogates: at least 20.2% of patients regularly consume moonshine and 11.8% of patients use surrogates, the most popular among which are medications with a high percentage of ethanol and industrial spirits. The pattern of samogon consumption as well as the pattern of vodka consumption is characterized by explosiveness. The social context of the use of samogon is practically not different from the context of the use of licensed vodka. 

The main sources of industrial alcohol are the so-called dual use of alcohol-containing liquids (household chemicals, disinfectants, and window washing fluid) with an ethyl alcohol content of 70%–96% by volume. After homemade neutralization of the chemical additives, industrial alcohol is diluted with water, bottled, and then sold. The price of the surrogate is one and one half to two times cheaper than state vodka, which explains its popularity among people who abuse alcohol. Hawthorn and Motherwort tinctures stand out as being two of the most popular of the nonbeverage alcohol drunk by alcohol dependent patients. These tinctures can be obtained without a prescription at practically any pharmacy and contain at least 65% alcohol by volume. Tincture of Hawthorn is sold in 100 mL bottles for $ 0,70 and tincture of Motherwort is sold in 50 mL bottles for $0,50. It is obvious that the high availability and affordability of industrial alcohol and medicines with high alcohol content are a main reason for its popularity among alcohol dependent population in Belarus. This situation requires an appropriate policy response including considerable enforcement efforts. 

Cheapness was quoted commonly as a reason for moonshine consumption [9, 19]. It appears, however, that for almost half of the respondents, the main motive for consuming samogon is the belief that it is a chemically purer product than the licensed vodka. These data contradict established notions that the main motive for consuming samogon is its low price. It is obvious that the sale of counterfeit products through the official distribution network discredits the quality of licensed alcohol and strengthens the confidence of consumers of alcohol in the fact than samogon is a chemically purer product that the licensed vodka. 

Accumulated research evidence suggests that affordability of alcohol is one of the most important predictors of alcohol consumption [20–22]. In relation to this it is reasonable to assume that heavy drinkers might be particularly sensitive to reduction in affordability of alcohol because they usually have a low income. The findings from this study related to alcohol control policy options suggest that alcohol dependent persons are sensitive to price changes of spirits (vodka) as the lowest-cost form of ethanol. These outcomes provide additional evidence that decrease in affordability of alcohol is an effective strategy for reducing alcohol-related harm. It should be noted, however, that one of the objections to pricing policy as a public health strategy is that dependent drinkers are likely to switch to surrogates in the face of licensed alcohol price increase [23]. Indeed, the results from present survey suggest that the high price of legal alcohol would increase samogon drinking. Therefore, any attempt to decrease alcohol affordability requires a degree of flexibility to tackle the problem of noncommercial alcohol.

In conclusion, this study explores types of alcohol and surrogates consumed, patterns of consumption, and reasons behind noncommercial alcohol consumption among alcohol dependent patients in Belarus and provides useful information that can be used for public health interventions. The results of this study suggest the existence of the problem of consumption of noncommercial alcohol among alcohol dependent patients, the most popular among which are SAMOGON, medications with a high percentage of ethanol and industrial spirits. The belief that, according to quality criteria, samogon exceeds licensed vodka is the main motive for its consumption. In this regard, it is urgent to inform the population about the potential risk to one's health from consuming surrogate alcohols. In addition, arrangements need to be taken to prevent the sale of counterfeit vodka through the government trade network. It should also be emphasized that the problem of noncommercial alcohols cannot be tackled without simultaneously taking steps to reduce the consumption of licensed beverages. The complex of these measures will allow the level of alcohol-related problems in society to be reduced. 

## Figures and Tables

**Table 1:** Selected sample background.

Sample characteristics	Percentage
Age (years)	39.7 ± 1.2
Marital status	
Single	56.8
Married	43.2
Education	
Primary	3.2
Secondary	77.4
High	19.5
Employment	
Unemployed	33.3
Manual professions	51.8
Nonmanual	8.7
Retirees	6.2
Income level	
Below average	43.3
Average	34.8
Above average	22.0
